# Complete sequences of KPC-2-encoding plasmid p628-KPC and CTX-M-55-encoding p628-CTXM coexisted in *Klebsiella pneumoniae*

**DOI:** 10.3389/fmicb.2015.00838

**Published:** 2015-08-19

**Authors:** Li Wang, Haihong Fang, Jiao Feng, Zhe Yin, Xiaofang Xie, Xueming Zhu, Jie Wang, Weijun Chen, Ruisheng Yang, Hong Du, Dongsheng Zhou

**Affiliations:** ^1^Department of Clinical Laboratory, The First Hospital Affiliated to Henan UniversityKaifeng, China; ^2^State Key Laboratory of Pathogen and Biosecurity, Beijing Institute of Microbiology and EpidemiologyBeijing, China; ^3^Department of Clinical Laboratory, The Second Affiliated Hospital of Soochow UniversitySuzhou, China; ^4^Beijing Institute of Genomics, Chinese Academy of SciencesBeijing, China

**Keywords:** *Klebsiella pneumoniae*, KPC-2, CTX-M-55, p628-KPC, p628-CTXM, promoter

## Abstract

A carbapenem-resistant *Klebsiella pneumoniae* strain 628 was isolated from a human case of intracranial infection in a Chinese teaching hospital. Strain 628 produces KPC-2 and CTX-M-55 encoded by two different conjugative plasmids, i.e., the IncFII_K_ plasmid p628-KPC and the IncI1 plasmid p628-CTXM respectively. *bla*_KPC−2_ is captured by a Tn*1722*-based unit transposon with a linear structure. ΔTn*3*-IS*Kpn27-bla*_KPC−2_-ΔIS*Kpn6*-ΔTn*1722* and this transposon together with a mercury resistance *(mer*) gene locus constitutes a 34 kb acquired drug-resistance region. *bla*_KPC−2_ has two transcription starts (nucleotides G and C located at 39 and 250 bp upstream of its coding region respectively) which correspond to two promoters, i.e., the intrinsic P1 and the upstream ISK*pn27*/Tn*3*-provided P2 with the core −35/−10 elements TAATCC/TTACAT and TTGACA/AATAAT respectively. *bla*_CTX−M−55_ is mobilized in an IS*Ecp1*-*bla*_CTX−M−55_-Δ*orf477* transposition unit and appears to be the sole drug-resistant determinant in p628-CTXM. *bla*_CTX−M−55_ possesses a single transcription start (nucleotides G located at 116 bp upstream of its coding region) corresponding to the IS*Ecp1*-provided P1 promoter with the core −35/−10 element TTGAAA/TACAAT. All the above detected promoters display a characteristic of constitutive expression. Coexistence of *bla*_KPC_ and *bla*_CTX−M_ in *K. pneumoniae* has been reported many times but this is the first report to gain deep insights into genetic platforms, promoters, and expression of the two coexisting *bla* genes with determination of entire nucleotide sequences of the two corresponding plasmids.

## Introduction

KPC-producing *Klebsiella pneumoniae* has spread worldwide and became an emerging pathogen with serious clinical and infection control implications (Tzouvelekis et al., 2012; Munoz-Price et al., 2013). Coexistence of *bla*_KPC_ and *bla*_CTX−M_ in *K. pneumoniae* has been reported in several countries, such as *bla*_KPC−2_/*bla*_CTX−M−1_ group, *bla*_KPC−2_/*bla*_CTX−M−2_ group, and *bla*_KPC−2_/*bla*_CTX−M−8_ group in Brazil (Peirano et al., 2009), *bla*_KPC−2_/*bla*_CTX−M−10_, *bla*_KPC−2_/*bla*_CTX−M−15_, and *bla*_KPC−3_/*bla*_CTX−M−2_ in Israel (Leavitt et al., 2007, 2010), *bla*_KPC−2_/*bla*_CTX−M−14_ in China (Cai et al., 2008), and *bla*_KPC−2_/*bla*_CTX−M−15_ in Greece (Souli et al., 2010). However, all these studies are confined to PCR detection and sequencing of *bla* genes, lacking deeper characterization of mechanisms of drug resistance. This study describes co-production of KPC-2 and CTX-M-55 in a clinical *K. pneumoniae* strain 628 from China. The *bla*_KPC−2_ and *bla*_CTX−M−55_genes are encoded by two different conjugative plasmids, p628-KPC and p628-CTXM respectively. The complete nucleotide sequences of p628-KPC and p628-CTXM are determined and then compared with other genetically closely related plasmids to gain deep insights into genetic structures of relevant plasmids and resistance gene loci. In addition, the promoters and their expression characteristics of these two plasmid-borne *bla* genes are dissected experimentally.

## Materials and methods

### Bacterial strains and identification

*K. pneumoniae* strain 628 was isolated from the cerebrospinal fluid specimen of a 64-year-old male with intracranial infection in a Chinese teaching hospital in October 2010. Bacterial species identification was performed using Bruker MALDI Biotyper (Bruker Daltonics, Bremen, Germany) and 16s rRNA gene sequencing (Frank et al., 2008). The major carbapenemase and extended-spectrum beta-lactamase (ESBL) genes were detected by PCR, followed by sequencing on an ABI Sequencer (Applied Biosystems, Foster City, CA, USA) (Chen et al., 2015). Bacterial antimicrobial susceptibility was tested by using VITEK 2 and judged by CLSI standard (CLSI, 2012).

### Plasmid transfer

Plasmid conjugal transfer experiments were carried out with *Escherichia coli* EC600 (LacZ^−^, Nal^R^, Rif^R^) being used as recipient and strain 628 as donor. Three milliliter of overnight culture of each of donor and recipient bacteria were mixed together, harvested and resuspended in 80 μl of Brain Heart Infusion broth (BD Biosciences, San Jose, CA, USA). The mixture was spotted on a 1 cm^2^ filter membrane that was placed on Brain Heart Infusion agar (BD Biosciences, San Jose, CA, USA) plate, and then incubated for mating at 37°C for 12 to 18 h. Bacteria were washed from filter membrane and spotted on Muller-Hinton agar (BD Biosciences, San Jose, CA, USA) plate containing 1000 mg/L rifampin (Merck, Darmstadt, Germany) and 200 mg/L ampicillin (Merck, Darmstadt, Germany) for selection of *bla*_CTX−M_- or *bla*_KPC_-positive *E. coli* transconjugants.

### Determination of plasmid DNA sequence

Plasmid DNA was isolated from the cell culture of *E. coli* transconjugant using Qiagen large construct kit (Qiagen, Hilden, Germany) and then sequenced by using whole-genome shotgun strategy in combination with Illumina HiSeq 2500 (Illumina, San Diego, CA, USA) sequencing technology. The contigs were assembled with Velvet and the gaps were filled through combinatorial PCR and Sanger Sequencing on an ABI Sequencer. The genes were predicted with GeneMarkS™ and further annotated by BLASTP and BLASTN against UniProt and NR databases.

### RNA isolation and primer extension assay

Bacteria were cultured overnight in Mueller-Hinton broth (BD Biosciences, San Jose, CA, USA). Total RNAs were extracted from harvested bacterial cells using TRIzol Reagent (Life Technologies, Carlsbad, CA, USA). RNA quality was monitored by agarose gel electrophoresis, and RNA quantity was determined by spectrophotometry. Each of the [γ-^32^P] ATP end-labeled primers GCTCAGTGGAACGAAAAC, AGCCGCCAAAGTCCTGTTCG, and CATGGGATTCCTTATTCTG, which corresponded to *bla*_KPC−2_ promoter P2, *bla*_KPC−2_ promoter P1, and *bla*_CTX−M−55_ promoter P1 respectively, was annealed with total RNA sample for primer extension assay as described previously (Zhang et al., 2011). For different cell cultures in a single experiment, equal amounts of total RNA were used as starting materials. The corresponding end-labeled primers were also used for sequencing the PCR amplicons generated by the primer pairs TCAGCGACATCGTCAACC/GGTCGTGTTTCCCTTTAGCC, TCAGGTGGCACTTTTCGG/GGTCGTGTTTCCCTTTAGCC, and AGACCTTTCGTTTGAAGTATG/AGCTTATTCATCGCCACGTT for *bla*_KPC−2_ promoter P2, *bla*_KPC−2_ promoter P1, and *bla*_CTX−M−55_ promoter P1 respectively. DNA sequencing was carried out using AccuPower & Top DNA Sequencing Kit (Bioneer, Daejeon, Korea). Primer extension products and sequencing materials were analyzed on 8 M urea-6% polyacrylamide gel electrophoresis. Radioactive species were detected by autoradiography.

### Nucleotide sequence accession numbers

The complete sequences of plasmids p628-KPC and p628-CTXM were submitted to GenBank under accession numbers KP987218 and KP987217 respectively.

## Results and discussion

### Characterization of *K. pneumoniae* strain 628

Strain 628 harbors *bla*_KPC−2_, *bla*_CTX−M−55_, *bla*_*SHV*_, and *bla*_*TEM*_. *bla*_KPC−2_ and *bla*_CTX−M−55_are located plasmids p628-KPC and p628-CTXM respectively. Conjugative transfer of p628-KPC or p628-CTXM into EC600 generates the transconjugant 628-KPC-EC600 (*bla*^+^_KPC−2_, *bla*^−^_CTX−M−55_, *bla*^−^_SHV_, and *bla*^−^_TEM_) or 628-CTXM-EC600 (*bla*^−^_KPC−2_, *bla*^+^_CTX−M−55_, *bla*^−^_SHV_, and *bla*^−^_TEM_) respectively. All of 628, 628-KPC-EC600 and 628-CTXM-EC600 are resistant to ampicillin, ampicillin/sulbactam, penicillin, monobactam, and cephalosporins tested (Table 1). 628 and 628-KPC-EC600 (but not 628-CTXM-EC600) are resistant to piperacillin/tazobactam. 628 and 628-KPC-EC600 (but not 628-CTXM-EC600) are carbapenem-resistant.

**Table 1:** **Antimicrobial drug susceptibility profiles**.

**Antibiotics**	**MIC (mg/L)/antimicrobial susceptibility**			
	**628**	**628-KPC-EC600**	**628-CTXM-EC600**	**EC600**
Ampicillin	≥32/R	≥32/R	≥32/R	16/I
Ampicillin/sulbactam	≥32/R	≥32/R	≥32/R	4/S
Piperacillin	≥128/R	≥128/R	≥128/R	≤4/S
Piperacillin/tazobactam	≥128/R	≥128/R	≤4/S	≤4/S
Aztreonam	≥64/R	≥64/R	≥64/R	≤1/S
Cefazolin	≥64/R	≥64/R	≥64/R	≤4/S
Cefuroxime sodium	≥64/R	≥64/R	≥64/R	16/I
Cefuroxime axetil	≥64/R	≥64/R	≥64/R	16/I
Ceftriaxone	≥64/R	≥64/R	≥64/R	≤1/S
Ceftazidime	≥64/R	16/R	≥64/R	≤1/S
Imipenem	≥16/R	≥16/R	≤1/S	≤1/S
Meropenem	≥16/R	2/R	≤0.25/S	≤0.25/S
Ciprofloxacin	≥4/R	≤0.25/S	≤0.25/S	≤0.25/S
Levofloxacin	≥8/R	0.5/S	0.5/S	0.5/S
Macrodantin	≥512/R	≤16/S	≤16/S	≤16/S
Amikacin	≤2/S	≤2/S	≤2/S	≤2/S
Tobramycin	≤1/S	≤1/S	≤1/S	≤1/S
Trimethoprim/sulfamethoxazole	40/S	≤20/S	≤20/S	≤20/S

### Complete nucleotide sequence of p628-KPC

**Figure 1:**
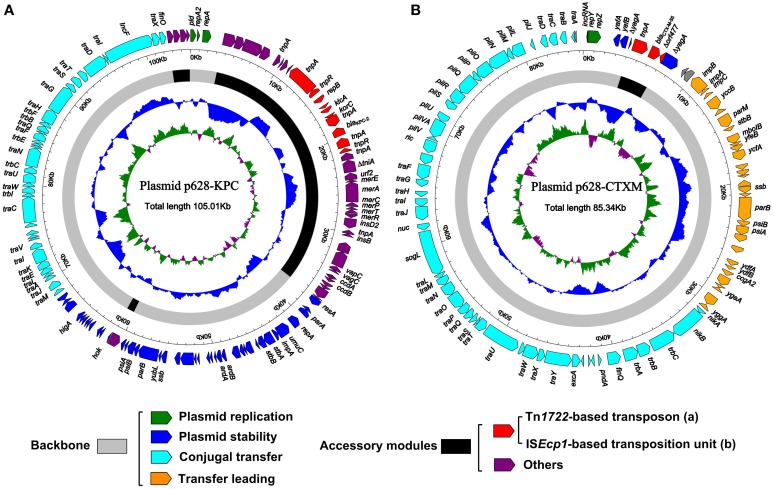
**Schematic maps of p628-KPC (A) and p628-CTXM (B)**. Genes are denoted by arrows and colored based on gene function classification. The innermost circle presents GC-Skew [(G-C)/(G+C)] with a window size of 500 bp and a step size of 20 bp. The blue circle presents GC content. Shown also are backbone and accessory module regions.

**Figure 2:**
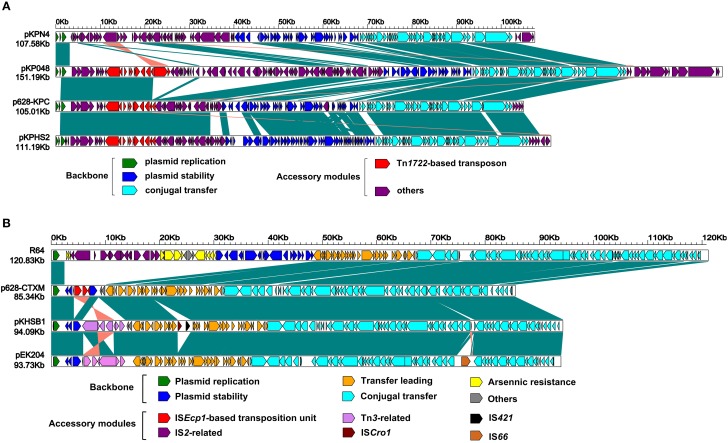
**Linear comparison of sequenced plasmids**. Genes are denoted by arrows and colored based on gene function classification. Shading regions denote regions of homology (>98% nucleotide similarity). Included are p628-KPC **(A)** and p628-CTXM **(B)** and their closely related plasmids.

The entire nucleotide sequence of p628-KPC is 105,008 bp in length, forming a circular plasmid with an average G+C content of 53.22 and a total of 127 open reading frames (ORFs) annotated (Figure 1A). p628-KPC belongs to the IncFII_K_ incompatibility group and harbors IncFII_K_ *repA* and the second IncFIB-like *repA2*, both of which encode replication initiation proteins. The p628-KPC backbone, 67,515 bp in length, is composed of DNA regions for plasmid replication (*repA* and *repA2*) and stability (*parAB, stbAB, ssb*, etc), and conjugal transfer (*tra, trb*, etc), which show >98% sequence identity to the corresponding regions of the IncFII_K_ plasmids pKPN4 (GenBank accession number CP000649), pKP048 (Jiang et al., 2010), and pKPHS2 (CP003224) (Figure 2A). The overall structure of p628-KPC is most similar to that of pKPHS2 (91% query coverage and 98% maximum nucleotide identity) (Figure 2A). pKPN4 is recovered from clinical *K. pneumoniae* MGH 78578 and represents the reference IncFII_K_ plasmid, carrying *bla*_SHV−12_ (cephalosporin resistance), and *aac(6*′*)* and *aadA* (aminoglycoside resistance). pKP048 and pKPHS2 are from two KPC-2-producing clinical *K. pneumoniae* isolates from China.

As shown in Figures 1A,2A, p628-KPC contains three distinct accessory modules: a 34 kb drug-resistance region, a 1038 bp IS*Kpn28*-based element, and a 2302 bp region of unknown function [identical sequences can be found in *bla*_KPC−2_-carrying plasmid pKPCAPSS (KP008371) and *qnrS1*-harboring pE66An (HF545433)]. The 34 kb region harbors two drug-resistance loci, the *mer* locus (mercury resistance) and the *bla*_KPC−2_ locus, and it is almost the same as the counterpart of pKPHS2 (Figure 2A). A 71 kb multi-drug-resistance region in pKP048 (Jiang et al., 2010) is composed of the 34 kb region of p628-KPC and the extra part (carrying *bla*_DHA−1_, *qnrB4*, and *armA* encoding resistance to cephalosporins, fluoroquinolones, and aminoglycosides respectively) absent from p628-KPC (Figure 2A).

### Complete nucleotide sequence of p628-CTXM

The p628-CTXM genome consists of an 85,338 bp circular DNA molecule with an average G+C content of 49.71 and harbors a total of 92 ORFs annotated (Figure 1B). p628-KPC belongs to the IncI1 incompatibility group expressing the replication initiation protein RepZ. The p628-CTXM backbone, 82,357 bp in length, contains DNA regions for plasmid replication (*repY, repZ*, and *inc*), conjugal transfer (*tra, trb, pil*, etc) and transfer leading (*imp, yfa* to *yfh, yga* to *ygg*, etc), which show >98% sequence identity to the corresponding regions of the IncI1 plasmids R64 from *Salmonella enterica* serovar Typhimurium (Sampei et al., 2010), pKHSB1 from *Shigella sonnei* (Holt et al., 2013) and pEK204 from *E. coli* O25:H4-ST131 clone (Woodford et al., 2009) (Figure 2B). Another backbone component is the plasmid stability region, composed of three genes *yafA, yafB*, and *yagA*, which is highly conserved among p628-CTXM, pKHSB1, and pEK204; by contrast, the corresponding segment of R64 is a 17.8 kb region which harbors at least 18 genes and especially include those encoding site-specific recombination (*resD* and *yefA*) and partition (*parAB*) of replicated DNA into daughter cells during cell division (Sampei et al., 2010) (Figure 2B).

R64 carries a single accessory module, a 17 kb IS*2*-based transposon, which interrupts *arsA1* (a member of the *arsR1-arsD1-arsA1-arsB-arsC* operon) (Sampei et al., 2010). p628-CTXM harbors a single accessory module, a 2980 bp IS*Ecp1*-related element, which interrupts *yagA* (a member of the plasmid stability region) (Figures 1B, 2B). Two distinct Tn*3*-related elements, 7935 and 8014 bp in length, are inserted downstream of *yagA* in pKHSB1 and pEK204 respectively. There are still additional accessory modules including IS*Cro1* and IS*421* for pKHSB1, and IS*66* for pEK204.

### Genetic surroundings of *bla*_*KPC-2*_

As characterized in European and American countries, the *bla*_KPC_ genes are located in a Tn*3*-family transposon named Tn*4401*, which is present on a wide variety of plasmids varying in size, structure and replicon (Naas et al., 2008; Kitchel et al., 2009, 2010; Chen et al., 2012; Bryant et al., 2013; Chmelnitsky et al., 2014). At least eight isoforms of Tn*4401* have been named, i.e., Tn*4401a* to Tn*4401g* and a separate Tn*4401d* (Table S1 in Supplementary Material). Several unnamed Tn*4401* isoforms have been also reported recently (Cuzon et al., 2011; Li et al., 2011; Ho et al., 2013b; Naas et al., 2013; Perez-Chaparro et al., 2014). Tn*4401b* is considered as the prototype one, and the other isoforms result from occurrence of distinct deletion or insertion events at different sites.

**Figure 3:**
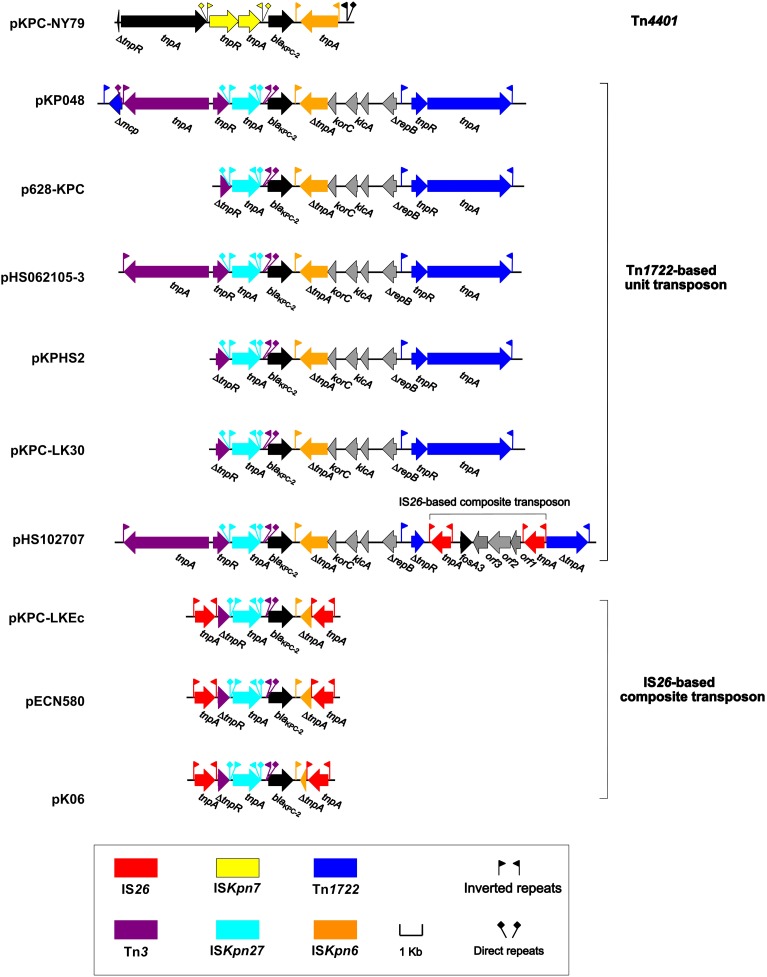
**Linear comparison of ***bla***_KPC−2_ genetic surroundings**. Genes are denoted by arrows and colored based on gene function classification.

As shown in Table 2 and Figure 3, the *bla*_KPC−2_ genetic environments from China can be assigned into three main categories: Tn*4401* with the IS*Kpn7*-*bla*_KPC−2_-IS*Kpn6* core structure (pKPC-NY79), the Tn*1722*-based unit transposons with the IS*Kpn27*-*bla*_KPC−2_-ΔIS*Kpn6* core structure [pKP048, p628-KPC, pHS062105-3, pKPHS2, pKPC-LK30, and pHS102707; IS*Kpn27* is initially named in the ISfinder database (Siguier et al., 2006)], and the IS*26*-based composite transposons with the IS*Kpn27*-*bla*_KPC−2_-ΔIS*Kpn6* core structure (pKPC-LKEc, pECN580, and pKo6). The Tn*4401* of pKPC-NY79 is a novel isoform of Tn*4401a* with *tnpR* truncated. The prototype Tn*1722*-based transposon as observed in pKP048 has a linear structure Δ*mcp*-Tn*3*-IS*Kpn27-bla*_KPC−2_**-ΔIS*Kpn6-korC-klcA*-unkown ORF**-Δ*repB*- Tn*1722*. Various truncations within the 5′ terminal Δ*mcp*-Tn*3* region can be identified for different KPC-encoding plasmids from China; in p628-KPC, a truncation within Δ*mcp*-Tn*3* leaves only a 402 bp remnant of the Tn*3 tnpR* gene at the 5′ end of Tn*1722*-based transposon. Interestingly, an IS*26*-based composite transposon, which is almost identical to the counterpart in pHK23 (recovered from pig-derived *E. coli* in China) and harbors the fosfomycin resistance gene *fosA3* (Ho et al., 2013a), is inserted into the *tnpRA* locus of Tn*1722* in pHS102707, leaving *tnpR* and *tnpA* truncated. The IS*26*-based *bla*_KPC−2_-carrying transposons have a basic linear structure IS*26*-ΔTn*3*-IS*Kpn27-bla*_KPC−2_**-ΔIS*Kpn6*-IS*26*, for which presence of two IS*26* elements at both ends truncates IS*Kpn6* and Tn*3*; notably, different lengths of truncated IS*Kpn6* can be observed for these IS*26*-based transposons from different plasmids.

**Table 2:** **Genetic surroundings of ***bla***_KPC−2_ from China**.

***bla***_**KPC−2**_ **Genetic environment**		**Plasmid**			**Bacterium**	**Host**	**References**
**Core structure**	**Transposon**	**Name**	**Incomparability group**	**Accession number**			
IS*Kpn*7−*bla*_KPC−2_–IS*Kpn6*	Tn*3*-based Tn*4401*	pKPC-NY79	IncX3	JX104759	*K. pneumoniae*	Human patient	Ho et al., 2013b
IS*Kpn*27−*bla*_KPC−2_–ΔIS*Kpn6*	Tn*1722*-based unit transposon^@^	pKP048	IncFII^*^_K_	FJ628167	*K. pneumoniae*	Human patient	Shen et al., 2009
		p628-KPC	IncFII_^*^K_	KP987218	*K. pneumoniae*	Human patient	This study
		pHS062105-3	IncP3	KF623109	*K. pneumoniae*	Human patient	NA
		pKPHS2	IncFII_^*^K_	CP003224	*K. pneumoniae*	NA	NA
		pKPC-LK30	IncFII_*K^&^*_	KC405622	*K. pneumoniae*	Human patient	Chen et al., 2014b
		pHS102707	Unknown	KF701335	*E. coli*	Human patient	Li et al., 2015
IS*Kpn27*-*bla*_KPC−2_–ΔIS*Kpn6*	IS*26*-based composite transposon^@^	pKPC-LKEc	IncI/IncN/RepFIC	KC788405	*E. coli*	Human patient	Chen et al., 2014b
		pECN580	IncN	KF914891	*E. coli*	Human patient	Chen et al., 2014a
		pKo6	IncN	KC958437	*K. pneumoniae*	NA	NA

*Included are all the bla_KPC_-carrying plasmids with determined genome sequences from China*.

@ *See reference (Roberts et al., 2008) for classification of transposons*.

* *In addition to the IncFII_K_ repA, the plasmid contains the second IncFIB-like repA2*.

& *This plasmid harbors a repB putative replication initiation region but, surprisingly, lacks the IncFII_K_ repA*.

### Genetic surroundings of *bla*_CTX−M−55_

**Figure 4:**
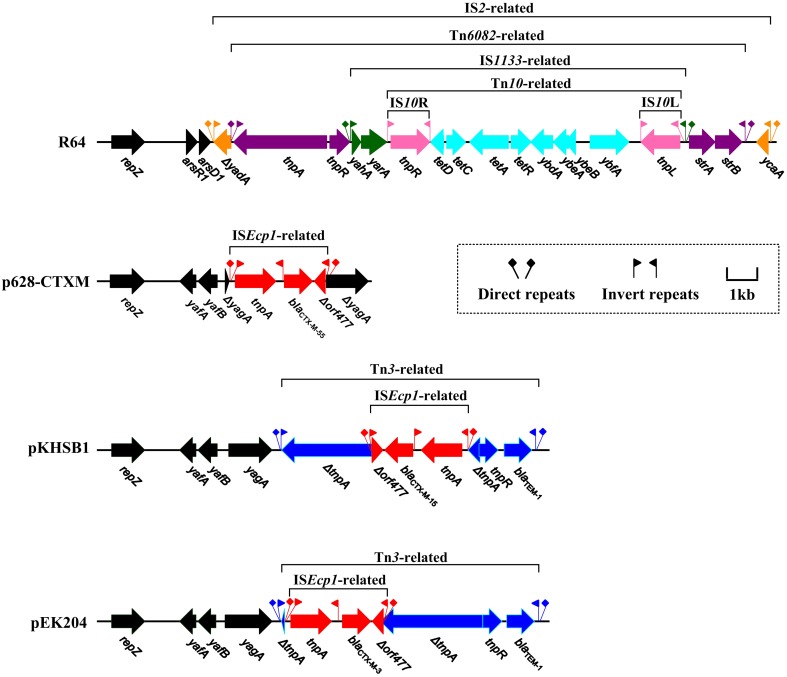
**Linear comparison of ***bla***_CTX−M_ genetic surroundings**. Genes are denoted by arrows and colored based on gene function classification.

R64, p628-CTXM, pKHSB1, and pEK204 carry a 17 kb IS*2*-based mobile element, a 2980 bp IS*Ecp1*-based transposition unit, a 7935 bp Tn*3*-based element, and an 8014 bp Tn*3*-based element respectively; each of them is the sole determinant for antibiotics resistance of the corresponding plasmid (Figure 4). For R64, stepwise insertions occur to eventually assemble the IS*2*-based element: insertion of IS*2* into *arsA1*, that of Tn*6082* into IS*2*, that of IS*1133* into Tn*6082*, and finally that of Tn*10* into IS*1133*; the *tet* locus carried by Tn*10* and the *strAB* operon carried by Tn*6082* account for resistance to tetracycline and streptomycin respectively.

A lot of *bla*_CTX−M−1_ group genes such as *bla*_CTX−M−55_, *bla*_CTX−M−15_ and *bla*_CTX−M−3_ are often connected with IS*Ecp1* (upstream; responsible for capture and mobilization of *bla*_CTX−M_) and Δ*orf477* (downstream), constituting an IS*Ecp1*-*bla*_CTX−M_-Δ*orf477* transposition unit (Lartigue et al., 2006; Zong et al., 2010). In p628-KPC, the plasmid backbone gene *yagA* is disrupted by IS*Ecp1*-*bla*_CTX−M−55_-Δ*orf477*. In pKHSB1 and pEK204, a *bla*_*TEM*−1_-carrying Tn*3* transposon is inserted at the site downstream of *yagA* and the Tn*3 tnpA* gene is further disrupted by IS*Ecp1*-*bla*_CTX−M−15_-Δ*orf477* and IS*Ecp1*-*bla*_CTX−M−3_-Δ*orf477*, respectively. In addition, these two inserted IS*Ecp1*-based structures differ from each other with respect to targeting sites and oriented directions (Figure 4).

### Expression of *bla*_KPC−2_ and *bla*_CTX−M−55_

Each of the *bla*_KPC−2_ genes in Tn*4401a, b, d, f*, and *g* has two transcription starts, i.e., nucleotides G and C located at 39 and 289 bp upstream of *bla*_KPC−2_, which correspond to the two promoters P1 and P2 (re-designated P2^*ISKpn*7^ herein) with core −35/−10 elements TAATCC/TTACAT and TTGACA/TATCTT respectively (Naas et al., 2012). By contrast, *bla*_KPC−2_ from Tn*4401c* or *e* has only P1, while P2^*ISKpn*7^ is absent due to presence of 215 or 255 bp deletion within *bla*_KPC−2_ upstream region respectively (Naas et al., 2012).

**Figure 5:**
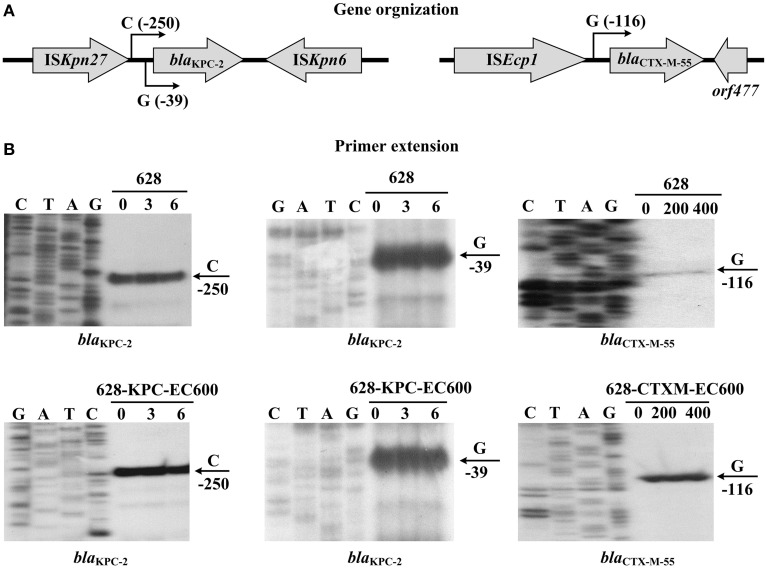
**Organization and expression of ***bla*** genes**. **(A)** Gene organization. Boxed arrows stand for length/direction of indicated genes. Broken-line arrows represent transcription starts for indicated *bla* genes. **(B)** Primer extension. Primer extension assay of the RNA transcript of each *bla* gene was performed for strain 628 cultured with addition of increasing amounts of imipenem or ampicillin. Lanes C, T, A, and G represent Sanger sequencing reactions. Lanes 0, 3, and 6 stand for 0, 3, and 6 mg/L imipenem. Lanes 0, 200, and 400 stand for 0, 200, and 400 mg/L ampicillin. Transcription start of each *bla* gene is indicated by arrow with nucleotide, and minus number under arrow indicates nucleotide position upstream of indicated *bla* gene. Representative data from at least two independent biological replicates are shown.

**Figure 6:**
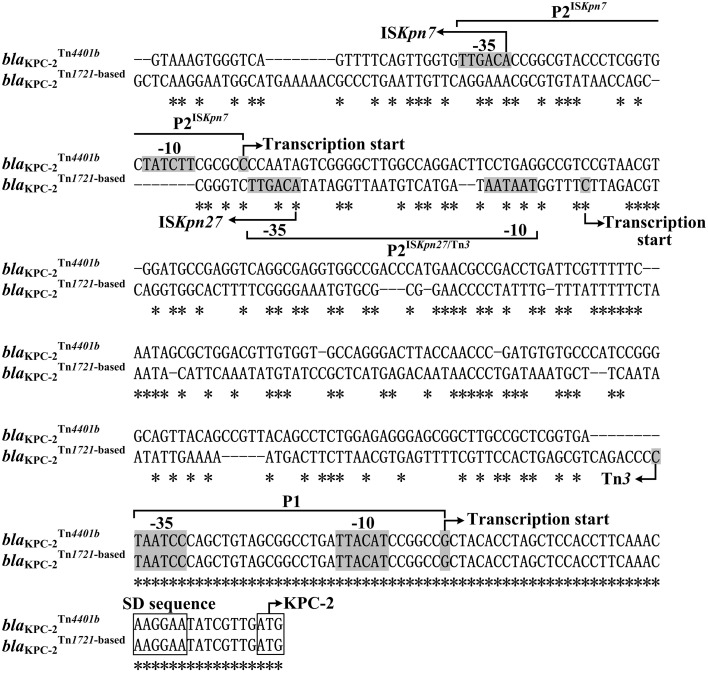
**Alignment of ***bla***_KPC−2_ upstream sequences**. The 357 bp upstream sequences together with the start codon of the *bla*_KPC−2_ genes were aligned by CLUSTALW (http://clustalw.ddbj.nig.ac.jp/). Shown were core promoter regions, −35 and −10 elements, transcription starts, Shine-Dalgarno (SD) sequences for ribosome recognition, and translation starts. Asterisks indicate the identical nucleotides.

In this work, the primer extension assay detected two transcription starts, i.e., nucleotides G and C located at 39 and 250 bp upstream of *bla*^Tn1722−*based*^_KPC−2_ from p628-KPC respectively; the corresponding two promoters were designated P1 and P2^*ISKpn*27∕*Tn*3^ with the core −35/−10 elements TAATCC/TTACAT and TTGACA/AATAAT respectively (Figures 5, 6). The first 74 bp fragments upstream of *bla*^Tn*4401b*^_KPC−2_ and *bla*^Tn*1722*^−based_KPC−2_ are essentially identical; the P1 promoter is located within this 74 bp region and thereby shared by *bla*^Tn*4401b*^_KPC−2_ and *bla*^Tn*1722*−based^_KPC−2_ (Figure 6). The next 280 bp region upstream of the above 74 bp fragment for *bla*^Tn*4401b*^_KPC−2_ is dramatically divergent at nucleotide level from the counterpart for *bla*^Tn*1722*−based^_KPC−2_; these two distinct 280 bp regions contain P2^IS*Kpn7*^ and P2^IS*Kpn*27∕Tn*3*^ respectively. The −35 element of P2^*IS*^^*Kpn*7^ is provided by IS*Kpn7* inserted at 319 bp upstream of *bla*^Tn^*4401b*_KPC−2_, while the −35 and −10 elements of P2^IS*Kpn27*∕Tn*3*^ are provided by IS*Kpn27* and Tn*3* inserted at 281 and 75 bp upstream of *bla*^Tn*1722*−based^_KPC−2_ respectively (Figure 6).

**Figure 7:**
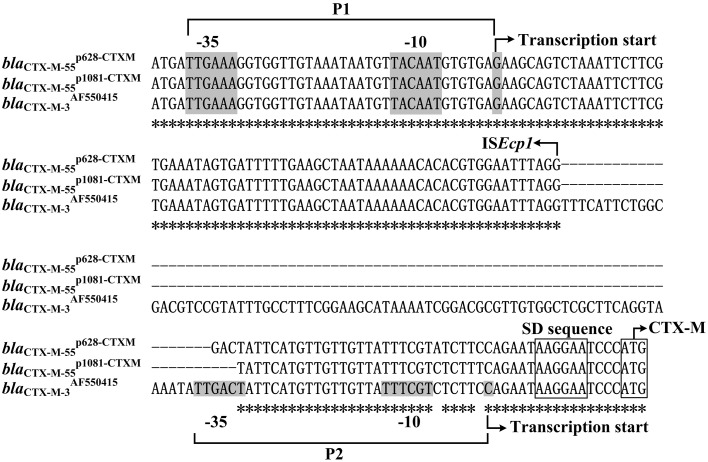
**Alignment of ***bla***_CTX−M_ upstream sequences**. The 153–235 bp upstream sequences together with the start codon of the *bla*_CTX−M_ genes were aligned by CLUSTALW. Shown were core promoter regions, −35 and −10 elements, transcription starts, SD sequences for ribosome recognition, and translation starts. Asterisks indicate the identical nucleotides

Spacer regions between IS*Ecp1* and *bla*_CTX−M−55_ from different IS*Ecp1*-*bla*_CTX−M−55_ isoforms display three different lengths, namely 45 bp (e.g., *bla*^p1081−CTXM^_CTX−M−55_) (Qu et al., 2014), 48 bp (e.g. *bla*^p628−CTXM^_CTX−M−55_), and 127 bp (e.g., *bla*^JQ343851^_CTX−M−55_). Two promoters, TTGAAA-N_18_-TACAAT-N_6_-G (organized as -35 element/-10 element/transcription start; named P1) and TTGACT-N_18_-TTTCGT-N_6_-C (P2), are experimentally identified for *bla*^AF550415^_CTX−M−3_ with a 127 bp spacer and moreover, the IS*Ecp1*-provided promoter P1 is stronger and more important than the intrinsic P2 promoter in the 127 bp spacer (Ma et al., 2011). The above result is applicable to the *bla*_CTX−M−55_ genes with the 127 bp spacer (Figure 7), because their IS*Ecp1*+spacer region is identical to the counterpart of *bla*^AF550415^_CTX−M−3_.

In the present study, the primer extension assays detected a transcription start, i.e., nucleotides G located at 116 bp upstream of *bla*_CTX−M−55_ (Figure 5), which corresponded to the P1 promoter shared by *bla*^p1081−CTXM^_CTX−M−55_ (Qu et al., 2014) and *bla*^p628−CTXM^_CTX−M−55_ (Figure 7). Compared with the 127 bp spacer, the 45 or 48 bp spacer for *bla*^p1081−CTXM^_CTX−M−55_ or *bla*^p628−CTXM^_CTX−M−55_ is a truncated form due to absence of a 82 or 79 bp region respectively. The deletion event impairs the −35 element of P2, most likely making the P2 activity undetectable for *bla*^p1081−CTXM^_CTX−M−55_ and *bla*^p628−CTXM^_CTX−M−55_ (Figure 7).

In addition, the primer extension assay showed that addition of increasing amounts of imipenem or ampicillin during cultivation of indicated strains 628, 628-KPC-EC600 and 628-CTXM-EC600 had no effect on activity of all the above promoters detected for *bla*_KPC−2_ or *bla*_CTX−M−55_, denoting constitutive expression of the above two resistance genes (Figure 5).

## Concluding remarks

KPC-2 and CTX-M-55 enzymes are produced by two different conjugative plasmids, p628-KPC and p628-CTXM respectively, in *K. pneumoniae* strain 628, and the sequences of these two plasmids are >98% identical to other relevant plasmids carrying the same resistance determinants previously sequenced. The detected *bla*_KPC−2_ gene is captured by a Tn*1722*-based unit transposon carried by an IncFII_*K*_-type multi-drug-resistant plasmid p628-KPC, and this gene has two different promoters, the intrinsic P1 and the IS*Kpn27*/Tn*3*-provided P2, both characteristic of constitutive expression. The detected *bla*_CTX−M−55_ gene, being the sole drug-resistant determinant in the plasmid, is mobilized in an IS*Ecp1*-based transposition unit carried by an IncI1 plasmid p628-CTXM, and this gene has a single IS*Ecp1*-provided promoter driving *bla*_CTX−M−55_ expression in a constitutive manner. Coexistence of *bla*_KPC_ and *bla*_CTX−M_ in *K. pneumoniae* has been reported many times, but this is the first report to gain deep insights into genetic platforms, promoters, and expression of the two coexisted *bla* genes. The IncFII_K_ and IncI1 plasmids have been frequently identified to carry horizontally acquired drug-resistant gene modules and could be transmitted across a number of bacterial species (Woodford et al., 2009; Jiang et al., 2010; Sampei et al., 2010; Holt et al., 2013), and increased surveillance of these drug-resistant plasmids is needed.

## Funding

This work is funded by National High-Tech Research and Development Program (2014AA021402), National Key Program for Infectious Disease of China (2013ZX10004216), and National Natural Science Foundation of China (31471184 and 31170127).

### Conflict of interest statement

The authors declare that the research was conducted in the absence of any commercial or financial relationships that could be construed as a potential conflict of interest.
