# Supplementary material for: Complete sequences of KPC-2-encoding plasmid p628-KPC and CTX-M-55-encoding p628-CTXM coexisted in Klebsiella pneumoniae
Source: Front Microbiol. 2015 Aug 19;6:838. doi: 10.3389/fmicb.2015.00838 (PMC4541600; doi:10.3389/fmicb.2015.00838)
Supplement: Supplementary file 1 [file Table1.DOCX]

**Table S1. Variants of *bla*_KPC_-carrying Tn*4401* elements**

| **Transposon** | **Genetic feature** | **Accession**  **number** | **Ref.** |
| --- | --- | --- | --- |
| Tn*4401a* | 99-bp deletion upstream of *bla*_KPC_ | EU176011 | [[1](#_ENREF_1)] |
| Tn*4401b* | no deletion | EU176014 | [[1](#_ENREF_1)] |
| Tn*4401c* | 215-bp deletion upstream of *bla*_KPC_ | DQ989640 | None |
| Tn*4401d* | 68-bp deletion upstream of *bla*_KPC_ | [HM769262](http://jac.oxfordjournals.org/external-ref?link_type=GEN&access_num=HM769262) | [[2](#_ENREF_2)] |
| Tn*4401d^&^* | a Tn4401b isoform with *tnpA* and *bla*_KPC_ truncated and IS*Kpn7* deleted | JN974188 | [[3](#_ENREF_3)] |
| Tn*4401e* | 255-bp deletion upstream of *bla*_KPC_ |  | [[4](#_ENREF_4)] |
| Tn*4401f* | a Tn4401b isoform with *tnpA* truncated and *tnpR* deleted | JQ837276 | [[5](#_ENREF_5)] |
| Tn*4401g* | a Tn4401b isoform with *tnpA* truncated due to insertion of a 256-bp sequence | KJ510411 | [[6](#_ENREF_6)] |

Tn*4401d^&^*: a separate designation of Tn*4401d*.

**References**

1. Naas T, Cuzon G, Villegas MV, Lartigue MF, Quinn JP, Nordmann P: **Genetic structures at the origin of acquisition of the beta-lactamase bla KPC gene**. *Antimicrob Agents Chemother* 2008, **52**(4):1257-1263.

2. Kitchel B, Rasheed JK, Endimiani A, Hujer AM, Anderson KF, Bonomo RA, Patel JB: **Genetic factors associated with elevated carbapenem resistance in KPC-producing Klebsiella pneumoniae**. *Antimicrob Agents Chemother* 2010, **54**(10):4201-4207.

3. Chen L, Chavda KD, Mediavilla JR, Jacobs MR, Levi MH, Bonomo RA, Kreiswirth BN: **Partial Excision of blaKPC from Tn4401 in Carbapenem-Resistant Klebsiella pneumoniae**. *Antimicrob Agents Chemother* 2012, **56**(3):1635-1638.

4. Kitchel B, Rasheed JK, Patel JB, Srinivasan A, Navon-Venezia S, Carmeli Y, Brolund A, Giske CG: **Molecular epidemiology of KPC-producing Klebsiella pneumoniae isolates in the United States: clonal expansion of multilocus sequence type 258**. *Antimicrob Agents Chemother* 2009, **53**(8):3365-3370.

5. Bryant KA, Van Schooneveld TC, Thapa I, Bastola D, Williams LO, Safranek TJ, Hinrichs SH, Rupp ME, Fey PD: **KPC-4 Is encoded within a truncated Tn4401 in an IncL/M plasmid, pNE1280, isolated from Enterobacter cloacae and Serratia marcescens**. *Antimicrob Agents Chemother* 2013, **57**(1):37-41.

6. Chmelnitsky I, Shklyar M, Leavitt A, Sadovsky E, Navon-Venezia S, Ben Dalak M, Edgar R, Carmeli Y: **Mix and match of KPC-2 encoding plasmids in Enterobacteriaceae-comparative genomics**. *Diagn Microbiol Infect Dis* 2014, **79**(2):255-260.
